# The Calcitonin/Calcitonin Gene-Related Peptide Family in Invertebrate Deuterostomes

**DOI:** 10.3389/fendo.2018.00695

**Published:** 2018-11-30

**Authors:** Toshio Sekiguchi

**Affiliations:** Noto Marine Laboratory, Division of Marine Environmental Studies, Institute of Nature and Environmental Technology, Kanazawa University, Kanazawa, Japan

**Keywords:** calcitonin/calcitonin gene-related peptide family, invertebrate deuterostome, evolution, amphioxus, ascidian

## Abstract

Calcitonin (CT)/CT gene-related peptide (CGRP) family peptides (CT/CGRP family peptides) including CT, CGRP, adrenomedullin, amylin, and CT receptor-stimulating peptide have been identified from various vertebrates and perform a variety of important physiological functions. These peptides bind to two types of receptors including CT receptor (CTR) and CTR-like receptor (CLR). Receptor recognition of CT/CGRP family peptides is determined by the heterodimer between CTR/CLR and receptor activity-modifying protein (RAMP). Comparative studies of the CT/CGRP family have been exclusively performed in vertebrates from teleost fishes to mammals and strongly manifest that the CGRP family system containing peptides, their receptors, and RAMPs was derived from a common ancestor. In addition, CT/CGRP family peptides and their receptors are also identified and inferred from various invertebrate species. However, the evolutionary process of the CT/CGRP family from invertebrates to vertebrates remains enigmatic. In this review, I principally summarize the CT/CGRP family peptides and their receptors in invertebrate deuterostomes, highlighting the study of invertebrate chordates including ascidians and amphioxi. The CT/CGRP family peptide that shows similar molecular structure and function with that of vertebrate CT has been identified from ascidian, *Ciona intestinalis*. Amphioxus, *Branchiostoma floridae* also possessed three CT/CGRP family peptides, one CTR/CLR receptor, and three RAMP-like proteins. The molecular function of the receptor complex formed by amphioxus CTR/CLR and a RAMP-like protein was clarified. Moreover, CT/CGRP family peptides have been identified in the superphylum Ambulacraria, which is close to Chordata. Finally, this review provides potential hypotheses of the evolution of CGRP family peptides and their receptors from invertebrates to vertebrates.

## Introduction

Calcitonin (CT)/CT gene-related peptide (CGRP) family peptides including CT, CGRP, adrenomedullin (AM), amylin (AMY), and CT receptor-stimulating peptide (CRSP) exhibit various functions in vertebrates such as the bone homeostasis, circulatory homeostasis, glucose metabolism, and feeding behavior. CT, discovered by Copp and Cheney in 1962 (1), represents the first identified CT/CGRP family peptide followed by the discovery of CGRP (1982) (2), AMY (1987) (3), AM (1993) (4), and CRSP (2003) (5). Although the numerous CT/CGRP family peptides possess multiple physiological functions, they share common molecular characteristics and the same receptor complex (6). Consistent with this, comparative genome analysis suggests that all CT/CGRP family peptide genes are originated from a small number of genes on ancestral proto-chromosomes (7). To date, comprehensive and partial representation of the CT/CGRP family peptide has been found from cyclostomes to mammals (7–13). In addition, the biological functions of CT/CGRP family peptides have been intensively investigated in mammals and teleosts (6, 7, 11–19). In addition, the molecular function of their receptors has been primarily studied in mammals. Overview of the ligand-receptor relationships between CT/CGRP family peptides and their corresponding receptors have been elucidated with a few exceptions in rodents and human (6, 7, 11–19). In teleost, this ligand-receptor relationship has been partially characterized (14, 20). Additionally, CT/CGRP family peptides have also been observed from protostomes to invertebrate deuterostomes (21–28). Therefore, CT/CGRP family peptides form a large peptide superfamily that exists extensively within bilaterians.

This review summarizes the basic and latest information of CT/CGRP family peptides and their receptors in deuterostomes including Chordata (vertebrates and protochordates), Ambulacraria, and Xenotubulla. I first introduce the CT/CGRP family peptides and their corresponding receptor recognition in vertebrates, with the subsequent focus in particular on the CT/CGRP family in protochordates including ascidians and amphioxi. Moreover, recent progress in the characterization of Ambulacraria CT/CGRP family peptides is also described. Finally, this review provides possible evolutionary scenarios of CT/CGRP family system from invertebrates to vertebrates.

## The CT/CGRP family in vertebrates

**Figure 1:**
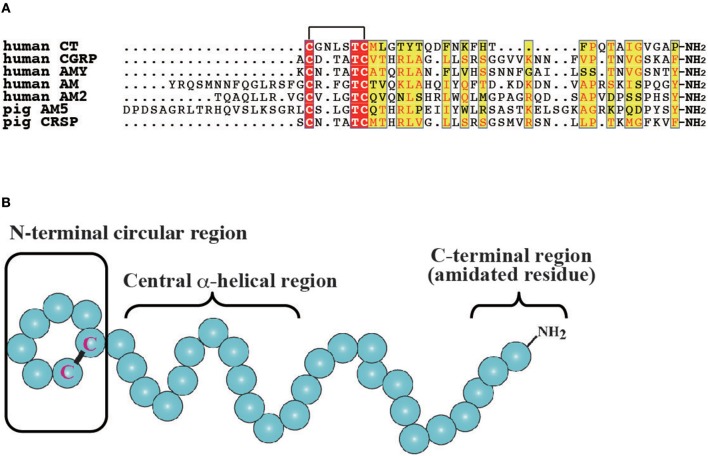
Molecular features of vertebrate CT/CGRP family peptides. **(A)** Primary structure of CT/CGRP family peptides in mammals. All identical and more than half of similar amino acid residues are denoted as a red and yellow box, respectively. The GenBank accession numbers of sequences are as follows: human CT, NP_001029124.1; human CGRP, CAA34070.1; human AMY, NP_001316130.1; human AM, AAC60642.1; human AM2, BAE46395.1; pig AM5, BAF64272.1; pig CRSP, NP_998907.1. Pig AM5 and CRSP were selected owing to the lack of human AM and CRSP sequences. The mature form of pig AM5 was predicted by using the signalP4.1 web server (http://www.cbs.dtu.dk/services/SignalP/). **(B)** The secondary structure of CT/CGRP family peptides. The N-terminal circular region, the central α-helical region, and the C-terminal amidated residue are conserved in vertebrates. The schematic diagram is based on the structure of CT.

The CT/CGRP family has been primarily investigated in mammals and teleosts. In mammals, CT comprises 32-amino acids and plays a role in bone homeostasis by repressing bone resorption through the inhibition of osteoclastic activity (Figure 1A) (18, 29). In turn, CGRP is a 37-amino acid neuropeptide that is expressed in the central and peripheral nervous systems and implicated in vasodilation (Figure 1A) (11). Moreover, CGRP is predominantly expressed in the sensory nerves and functions as a pain transmitter as well as a vasodilator (11). Two *CGRP* genes, designated as CGRP α and β, have been identified in mammals (11, 30). The gene for CGRPα (*CALCA*) synthesizes mRNA encoding CT and CGRP by tissue dependent alternative splicing, which are expressed in C cells of the thyroid gland and neural tissues, respectively (2, 31). In contrast, the gene for CGRPβ (*CALCB*) generates only CGRP peptide (11, 30). Mature peptides of CGRPα exhibit high amino acid sequence identity with CGRPβ (e.g., 92% identity in human CGRPα and β) (11). Mammals also have three AM paralogous genes, encoding AM, AM2/intermedin (AM2/IMD), and AM5 (Figure 1A) (4, 32–34). Although *AM5* gene has been observed in Artiodactyla and Carnivora (7), *AM5* gene is absent in rodents. In human and chimpanzee, a 2 bp deletion in the AM5 gene results in a frameshift mutation (7). AM exhibits various functions including vasodilation, hypotension, angiogenesis, and body fluid homeostasis (13, 35, 36). AMY is a 37-amino acid peptide that is released from islet β cells together with insulin when blood glucose levels increase (Figure 1A). AMY elicits the decrease of blood glucose level and loss of body weight through the suppression of glucagon release, food intake, and gastric emptying (12). CRSP is 37 or 38-amino acid peptide that is observed in Laurasiatheria such as cattle, dogs, goats, sheep, and horses, and is believed to be involved in food intake (Figure 1A) (5, 16). Notably, there are three CRSP isoforms in porcine (16), with these species predominantly lacking the gene for CGRPβ in favor of the *CRSP* gene (7). Moreover, comparative genome analysis indicates that *CRSP* gene is orthologous to *CGRP*β gene (7). Thus, mammalian CT/CGRP family peptides consist of multiple peptides possessing different biological activities.

In teleosts, the number of CT/CGRP family peptide members is larger than that of mammals, owing to a third-round whole genomic duplication (3RWGD) (7). There are two *CGRP* genes, referred to as *CGRP1* and *CGRP2*, which encode CT and CGRP in different exons as with the mammalian *CGRP*α gene, giving rise to mRNA for CT and CGRP by alternative splicing in the ultimobranchial gland and central and peripheral nervous systems, respectively. The association of CT activity with calcium homeostasis remains controversial in teleosts, unlike mammals (37). CGRP acts as a vasodilator and is likely to be involved in osmotic and ionic regulation (38–40). Teleost fish (e.g., medaka, zebrafish, and Japanese pufferfish) possess five AMs (7, 41). Teleost AMs also exhibit cardiovascular function and osmoregulatory activity (15, 34). Notably, AM2 and 5 are more potent than AM1 in teleost fish (15, 34). An AMY has also been reported in teleost fish (7, 42); however, no paralogous gene has been reported. *CRSP* gene has not been extracted from the teleost fish genome. Altogether, findings of mammalian and teleost CT/CGRP family peptides reveal that they demonstrate variable distribution and possess multiple physiological functions in vertebrates.

Comparisons of each CT/CGRP family peptide members in the same vertebrate spices show low primary amino acid sequence identity, whereas these peptides share common secondary amino acid structures such as the N-terminal circular structure formed by a disulfide bond, α-helical structure following the N-terminal region, and an amidated residue in the C-terminus (Figure 1B) (35, 43, 44). Moreover, synteny analysis of teleost and mammalian CT/CGRP family peptides suggests that CT/CGRP family peptide genes are derived from a common ancestral gene and diversified in teleost and mammalian lineages through genome duplication and rearrangement (7).

CT/CGRP family peptides function via two types of receptors containing the CT receptor (CTR) and CTR-like receptor (CLR) (19), which belong to secretin type (or family B) G-protein coupled receptors (GPCR). CTR and CLR has a long N-terminal extracellular domain that is involved in peptide binding (19). Molecular phylogenetic analysis of vertebrate CTR/CLR demonstrated that the *CTR* gene is paralogous to *CLR* gene (45). Moreover, peptide-receptor recognition is determined by the heterodimer between CTR/CLR and receptor activity-modifying protein (RAMP) (6). RAMP is a single-pass transmembrane protein that has four Cys residues which are conserved in vertebrates (46, 47). In mammals, a CTR, a CLR, and three RAMPs have been observed (19, 48). The heterodimer of CLR and RAMP1 generates a CGRP receptor (19, 48). In turn, the AM1 receptor is formed by the complex of CLR and RAMP2/3 (19, 48), whereas the existence of an AM2/IMD preferential receptor remains unclear (19). The AMY receptor consists of the heterodimer of CTR and RAMP1–3. Of interest, CGRP and AMY possess the same potency with regard to the CTR and RAMP1 complex (19). In contrast, both CT and CRSP activate CTR, with CRSP being more potent than CT (16).

Multiple CLRs and RAMPs have been observed in teleost fish (14). Mefugu, *Takifugu obscurus* possesses three CLRs and five RAMPs (14). Mefugu RAMPs have been divided into three groups including RAMP1/4, RAMP2/5, and RAMP3 by molecular phylogenetic analysis (14). The ligand-receptor relationship between CT/CGRP family peptides and their receptor complexes in teleosts is likely to be similar to that in mammals. For example, mefugu RAMPs are associated with the ligand selectivity of mefugu CLRs and CTR (14, 20). The heterodimer of mefugu CLR1 and mefugu RAMP1/4 forms the CGRP receptor. AM paralogs are recognized by the following combinations of mefugu CLR and RAMPs: CLR1-RAMP2/3/5 (AM1), CLR2-RAMP2 (AM1), CLR3-RAMP3 (AM2), and CLR1-RAMP3 (AM5). In addition, salmon CT recognizes mefugu CTR and human AMY can activate the complex of mefugu CTR-RAMP1–5 (14, 20). Consequently, the various physiological functions of CT/CGRP family peptides are determined by the complex combination of the CT/CGRP family peptide, its receptor (CTR or CLR), and RAMP in both mammals and teleosts.

Furthermore, RAMPs are also involved in the cell surface trafficking of CLR. The solo CLR is not transported from the endoplasmic reticulum (ER) to the golgi apparatus, whereas the binding of RAMP to CLR in the ER helps to translocate the CLR-RAMP complex to the cell surface. The cell surface trafficking activity of RAMP is conserved from teleosts to mammals (6, 49). Taken together, these findings suggest that the ligand-receptor system formed by CT/CGRP family peptides, their receptors, and RAMPs is conserved in vertebrates.

## CT/CGRP family peptide and its putative receptor in ascidians

**Figure 2:**
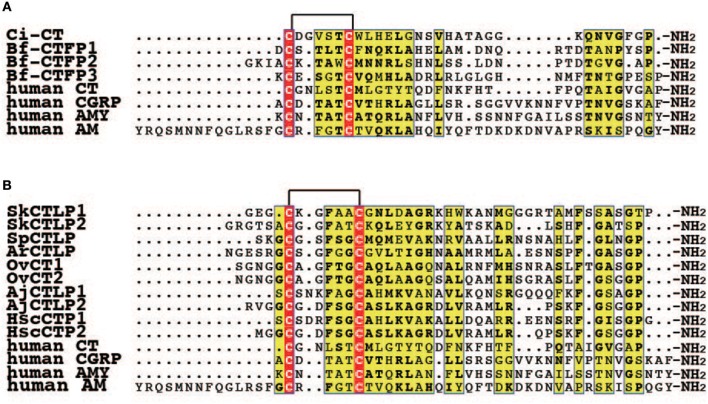
Multiple alignment of CT/CGRP family peptides in invertebrate deuterostomes. **(A)** Multiple alignment of CT/CGRP family peptides of protochordates and human. The GenBank accession numbers of sequences are as follows: Ci-CT, BAI63095.1; Bf-CTFP1, BAU51800.1; Bf-CTFP2, BAU51801.1; Bf-CTFP3, BAU51802.1; human CT, NP_001029124.1; human CGRP, CAA34070.1; human AMY, NP_001316130.1; human AM, AAC60642.1. **(B)** Multiple alignment of CT/CGRP family peptides of Ambulacraria and human. Accession numbers for the corresponding precursors are as follows: SpCTLP, XP_001177490.1 (GenBank); ArCTP, ALJ99957.1 (GenBank); OvCT1 and 2, MF15231 and MF15235 (GenBank). Amino acid sequences of SkCTLP1-2, AjCTLP1-2, and HscCTP1-2 were obtained from the following publications: SkCTLP1-2, Rowe et al. (25); AjCTLP1-2, Rowe et al. (25); HscCTP1-2, Suwansa-Ard et al. (36).

Ascidians are regarded as an extant invertebrate group that is the closest to vertebrates (50, 51). In the ascidian *Ciona intestinalis*, Sekiguchi et al. (21) cloned a CT/CGRP family peptide, designated as *Ciona* CT (Ci-CT), and evaluated its molecular features. Ci-CT consist of 30-amino acid residues that possesses the N-terminal circular region formed by a disulfide bond between Cys1 and Cys7 along with an amidated Pro residue in the C-terminus, which is similar to vertebrate CTs (Figure 2) (21). Although most vertebrate *CGRP* genes have a six exon and five intron structure, the gene for Ci-CT is composed of four exons and three introns. Moreover, it encodes only the Ci-CT peptide (21), rather than being alternatively spliced to generate the mRNA for CT and CGRP as for mammalian *CGRP*α gene (2). Consequently, the gene structure of Ci-CT is simple, suggesting that the complexity of the vertebrate *CGRP* gene was subsequently acquired during the process of vertebrate evolution.

Considering that the central region following the N-terminal ring structure in vertebrate CT/CGRP family peptides results in the formation of an α helix structure in lipophilic conditions such as the plasma membrane (52–54), the secondary structure of Ci-CT was measured using circular dichroism analysis. Titration of sodium dodecyl sulfate, which serves as a mimic of the plasma membrane, induced an α-helical structure in Ci-CT as well as salmon CT, suggesting that Ci-CT has a similar secondary structure as salmon CT (21). Moreover, Ci-CT also possesses vertebrate CT-like activity, as shown by its ability to evoke cAMP accumulation in COS-7 cells expressed by human CTR (21). Similar CT-like activity was also observed in an analysis using goldfish scales. The scales of teleost fishes are a dermal skeleton and contain osteoblasts and osteoclasts similar to mammalian bone (55, 56). Osteoblasts and osteoclasts are implicated in bone formation and bone resorption in response to calcemic hormone, respectively (57, 58). In the scale culture system, salmon CT significantly suppressed the osteoclastic activity, with a similar activity being detected upon Ci-CT treatment (21).

A *Ciona* gene that shows similarity with vertebrate CTR/CLR has also been identified by expressed sequence tag (EST) analysis of the neural complex (59). Molecular phylogenetic analysis revealed that the deduced *Ciona* CTR/CLR protein was located at the same clade as vertebrate CTR/CLR, suggesting that this protein is likely to be Ci-CT receptor (Ci-CT-R) (21). On the other hand, no RAMP ortholog has been detected in the *Ciona* genome by homology search (21). The *RAMP* gene is thus likely to have been lost in the *Ciona* lineage because large-scale gene loss (60) was predicted to occur in the ascidian lineage. However, it remains a possibility that homology searches failed to detect a *RAMP* ortholog gene, given that the amino acid sequence similarity of RAMP is low among species. Moreover, the receptor activity of Ci-CT-R has not yet been clarified, due to the lack of cell surface expression in mammalian and insect cell lines (21).

The physiological function of Ci-CT has not yet been evaluated, whereas Ci-CT expression analysis has been performed. *In situ* hybridization analysis of the Ci-CT mRNA in *Ciona* juveniles demonstrated that it could be detected in the neural complex, gill, digestive tract, blood cells, and endostyle (21). In addition, mRNA localization analysis for Ci-CT in the adult neural complex revealed that this mRNA is exclusively expressed in the neural gland (21), which is a non-neuronal tissue and is expected to be an exocrine gland. Therefore, Ci-CT is likely to act as a paracrine peptide and/or endocrine peptide. Further study of Ci-CT biological activity in *C. intestinalis* would therefore lay the foundation for assessing the functional evolution of chordate CT/CGRP family peptides.

Consequently, investigation of Ci-CT revealed that Ci-CT possesses CT-like properties including primary and secondary structure and biological activity. However, physiological function and genuine receptor of Ci-CT remain unclear. Therefore, although it is highly possible that Ci-CT belongs to a member of the CT/CGRP family peptides, it is difficult to define the orthology between Ci-CT and vertebrate CT.

## CT/CGRP family peptides and their genuine receptor complexes in amphioxi

Amphioxi are considered to be the most basal member in extant chordates (51, 61). Three CT/CGRP family peptides have been identified from the amphioxus, *Branchiostoma floridae* and designated as *B. floridae* CT/CGRP family peptides (Bf-CTFPs) (26). These genes are located on the same scaffold in the *B. floridae* genome database (Sekiguchi T. unpublished data). Bf-CTFP1, 2, and 3 comprise 31, 33, and 33-amino acid residues, respectively. All Bf-CTFPs contain the C-terminal Pro-amide, which is conserved in vertebrate CT (Figure 2) (26). However, the primary sequences of Bf-CTFPs are also reminiscent of CGRP, AM, AMY, and CRSP in terms of the amino acid length of the N-terminal circular region and the N-terminal extension flanked by a circular domain (26). Therefore, Bf-CTFPs possess hybrid features of the vertebrate CT/CGRP family in terms of primary structure.

To evaluate the secondary structure of Bf-CTFPs, a homology modeling study was performed using Bf-CTFPs, eel CT, salmon CT, human CT, and mutated salmon CT, which has a longer α-helical region and exhibits lower potency and affinity than wild-type salmon CT (62). This study revealed that Bf-CTFP1, 2, and 3 contained α-helical structure in the central region following an N-terminal circular region. The length and location of the α-helical structure of Bf-CTFPs was closer to that of eel CT and salmon CT than that of human CT and mutated salmon CT (62), which is in good agreement with the biological effects of Bf-CTFPs on vertebrates. In cultured goldfish scales, treatment of Bf-CTFPs repressed the osteoclastic activity similar to salmon CT. Moreover, this activity was antagonized by treatment with an N-terminal circular structure-truncated salmon CT. These findings strongly suggest that Bf-CTFPs bind to the goldfish CTR and function in osteoclastic cells, and further indicate that Bf-CTFPs share a similar secondary structure and biological activity with vertebrate CT (62). However, this structure-activity data should be carefully interpreted, as the α-helical structure in the central region of the peptide represents a common feature of CT/CGRP family peptides rather than a specific feature of CT. Thus, these findings strongly support that Bf-CTFPs are possible members of CT/CGRP family peptides in chordates. Furthermore, expression analysis of Bf-CTFPs using *in situ* hybridization revealed that the all Bf-CTFPs mRNAs were localized to the rostral part of the central nervous system, and Bf-CTFP2 and 3 were expressed in the midgut and hindgut, respectively (26). These findings suggest that Bf-CTFPs act as brain/gut peptides.

Candidates for a Bf-CTFP receptor (Bf-CTFP-R) and three RAMP-like proteins (Bf-RMAP-LPs) have also been cloned from adult amphioxus cDNA (26). Molecular phylogenetic analysis of chordate CTRs and CLRs indicated that Bf-CTFP-R is included in the same group as vertebrate CTR and CLR with high bootstrap support (26). Alignment of Bf-CTFP-R and human CTR/CLR revealed that Bf-CTFP-R exhibits 61.8 and 64.3% sequence similarity with the human CTR and CLR, respectively (26). Notably, Bf-RAMP-LPs possess a predicted transmembrane domain and four Cys residues that are conserved in the vertebrate RAMPs. Comparison of amino acid sequence of Bf-RAMP-LPs with human RAMP1–3 demonstrated that the three RAMP-LPs exhibit low amino acid sequence similarity (15.4–39.3%) (26). Consistent with this, molecular phylogenetic analysis of chordate RAMPs disclosed that the three Bf-RAMP-LPs diverged independently in the amphioxus lineage (26).

The function of Bf-RAMP-LPs as an escort protein has been evaluated by fluorescence activated cell sorting analysis (26). An N-terminal V5-tagged Bf-CTFP-R transfected into COS-7 cells was not detected by a V5 antibody without detergent, indicating that Bf-CTFP-R is not able to localize on the cell surface. In contrast, co-expression of wild-type Bf-RAMP-LPs with V5-tagged Bf-CTFP-R induced the translocation of V5-tagged Bf-CTFP-R to the cell membrane (26). These results strongly suggest that Bf-RAMP-LP acts as an escort protein and that Bf-CTFP-R is a RAMP-dependent receptor with regard to cell surface localization, similar to vertebrate CLR (26). The molecular function of the Bf-CTFP-R-Bf-RAMP-LP complex has also been uncovered. The ligand-receptor assay demonstrated that all Bf-CTFPs were not able to activate COS-7 cells expressed by Bf-CTFP-R. Moreover, Bf-CTFPs activated the COS-7 cells transfected with Bf-CTFP-R in conjunction with each Bf-RAMP-LPs in a dose-dependent manner (26). Comparison of the pEC_50_ values of the receptor complexes revealed that the order of potency of Bf-CTFPs depended on each Bf-RAMP-LP as follows: Bf-CTFP2 > Bf-CTFP3 = Bf-CTFP1 in Bf-CTFP-R-Bf-RAMP-LP1, Bf-CTFP2 = Bf-CTFP3 > Bf-CTFP1 in Bf-CTFP-R-Bf-RAMP-LP2; and Bf-CTFP2 > Bf-CTFP3 = Bf-CTFP1 in Bf-CTFP-R-Bf-RAMP-LP3 (26).

Altogether, these findings demonstrate that Bf-CTFPs are authentic ligands of the Bf-CTFP-R-Bf-RAMP-LP complexes, and that Bf-CTFP-R functions as a RAMP-dependent receptor similar to vertebrate CLR. Moreover, Bf-RAMP-LPs could be implicated in the formation of active receptor complex, in the trafficking of Bf-CTFP-R, and in the Bf-CTFPs selectivity of Bf-CTFP-R.

## The CT/CGRP family in ambulacraria

Ambulacraria, including hemichordates and echinoderms, constitutes a sister group to the chordates in deuterostomes (63). Recent genomic, and transcriptomic research has described the CT/CGRP family peptides in the Ambulacraria (22, 24, 25, 27, 28, 36). For example, in hemichordates, the acorn worm (*Saccoglossus kowalevskii*) possesses two CT/CGRP family peptides according to the genomic database (24, 25) (Figure 2). The first discovery of a CT/CGRP family peptide in echinoderms, a phylum consisting of crinoids, asteroids, ophiuroids, sea urchin, and sea cucumber, was obtained from a radial nerve and larval cDNA library in the sea urchin, *Strongylocentrotus purpuratus* (22). It comprised 37-amino acid residues designated as *S. purpuratus* CT-like peptide (SpCTLP). Furthermore, a 40-amino acid CT-type neuropeptide (CTP) was identified from transcriptome data of radial nerve cords in the Asteroidea, *Asterias rubens* (27). In *A. rubens*, larval localization of CTP elucidated by whole mount *in situ* hybridization has been reported (64). *A. rubens* CTP (ArCTP) mRNA was distributed in the adhesive disk and adjacent tissue in the brachiolariae stage (64). The existence of a CT/CGRP family peptide has also been reported based on transcriptomic information of three ophiuroid species (*Ophionotus victoriae, Amphiura filiformis*, and *Ophiopsila aranea*) (28). *A. filiformis* and *O. aranea* possess two transcriptional variants of a CT-type peptide precursor (28), with variant 1 and variant 2 including CT2 and CT1/2 peptides, respectively; however, *O. victoriae* lacks variant 1 (28). Moreover, a CT/CGRP family peptide of sea cucumber has been reported from two species (25, 36). A comprehensive peptide search of a cDNA library including different developmental stages and adult organs in the sea cucumber, *Apostichopus japonicus*, detected a transcript encoding a CT/CGRP family peptide precursor, termed *A. japonicus* CT-like peptide precursor (AjCTLPP) (25). The deduced amino acid sequence of AjCTLPP contains AjCTLP1 and AjCTLP2 peptides of 35 and 37-amino acid residues, respectively (25). In another sea cucumber, *Holothuria scabra, two* CT-type peptide precursor (CTPP) isoforms, designated as *H. scabra* CTPP long (HscCTPP-long) and HscCTPP-short, were detected from both neural and gonad cDNA library (36). The HscCTPP-long and short isoform contained both the 35-amino acid CT-type peptide 1 (CTP1) and 36-amino acid CTP2, and only CTP2, respectively (36). Reverse transcription-polymerase chain reaction (RT-PCR) analysis demonstrated that HscCTPP-long and short mRNAs are distributed in the circumoral nerve ring, radial nerve cord, longitudinal muscle, and intestine (36).

Overall, the primary amino acid sequence of Ambulacraria peptides resembles that of vertebrate CT (Figure 2). All Ambulacraria CT/CGRP family peptides and vertebrate CTs share the common primary structures of N-terminal circular region amino acid length and the C-terminal amidated Pro residue (Figure 2), except for only AjCTLP1, which has one amino acid longer circular region than that of vertebrate CTs (Figure 2). In contrast, the extension connecting the N-terminal circular region of Ambulacraria CT/CGRP family peptides is reminiscent of those of vertebrate CGRP, AM, AMY, and CRSP. Collectively, these observations suggest that Ambulacraria CT/CGRP family peptides also exhibit hybrid features of the various vertebrate CT/CGRP peptide family members.

Finally, although the presence of a CTR/CLR gene has been reported in acorn worm and sea urchin by genome search (24, 65), the ligand-receptor relationship between the CT/CGRP family peptide and CTR/CLR has not yet been reported in Ambulacraria. Further study will be required to elucidate the evolutionary process of CT/CGRP family peptide receptors during invertebrate deuterostome evolution.

## Possible evolutionary process of CT/CGRP family system from invertebrates to vertebrates

**Figure 3:**
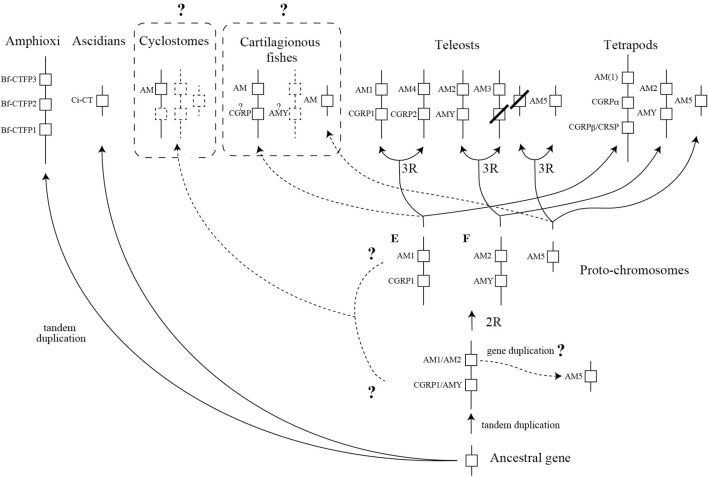
A schema predicting the diversification process of the CT/CGRP family peptide gene during chordate evolution. This schema is described as a modification of Takei's report (66). The dotted line and dotted box represent uncertain pathway and unidentified gene, respectively. Question mark on dotted line depicts the ambiguous pathway. Dotted boxes with a question mark exhibit that comprehensive identification of cartilaginous fish and cyclostome CT/CGRP family peptide is incomplete. 2R and 3R show second-round whole genome duplication and third-round whole genome duplication, respectively. CT, calcitonin; CGRP, CT gene-related peptide, AM, adrenomedullin; AMY, amylin; CRSP, CT receptor-stimulating peptide.

Accumulation of the data of CT/CGRP family from vertebrates and invertebrate chordates allows us to describe the evolutionary scenario of CT/CGRP family peptide. Given that Takei et al. reported a splendid evolutionary model of CT/CGRP family peptide gene in vertebrates (66), I propose an evolutionary scenario of CT/CGRP family peptide in chordates based on the Takei's model (Figure 3).

It is presumed that a common chordate ancestor possessed a single CT/CGRP family peptide gene (Figure 3). As shown above, three amphioxus CT/CGRP family peptide (*Bf-CTFP*) genes are tandemly arranged on the same chromosome, suggesting that amphioxus lineage-specific gene duplication occurred (Figure 3). On the other hand, ascidian CT/CGRP family peptide (*Ci-CT*) gene might be derived from the ancestral gene without duplication event.

After the occurrence of vertebrates, an ancestral gene might tandemly duplicate and form two genes, which are ancestral *AM1*/*AM2* gene and *CGRP*/*AMY* gene (Figure 3). *AM5* gene ancestor might be generated from an ancestral *AM1*/*AM2* gene by gene duplication (Figure 3). There is a widely accepted theory that second-round WGD (2RWGD) occurred after the appearance of vertebrates (67). Although 2RWGD is considered to occur before or after the emergence of cyclostome ancestor, the conclusion remains unclear (68–70). Cyclostomes are the basal vertebrates, which are the jawless animals and comprise lamprey and hagfish (71). *AM* gene has been identified in lamprey and hagfish (9). However, the other CT/CGRP family members have not been reported. Therefore, diversification process of CT/CGRP family peptide gene in extant cyclostomes from ancestral vertebrates remains to be clarified. Further study using currently available genomic information of lamprey and hagfish is necessary.

The comparative genome analysis of teleost fishes and tetrapods demonstrated that CT/CGRP family peptide gene located on the three proto-chromosomes, which is predicted by analyzing medaka genome (7, 72). In the common ancestor of teleosts and tetrapods, *AM1* and *CGRP* gene would be located on the proto-chromosome E. Moreover, *AM2* and *AMY* gene would be encoded on the proto-chromosome F. Localization of *AM5* gene is likely to be on unidentified chromosome except for proto-chromosome E and F. In teleost lineage, 3RWGD is expected to occur. The 3RWGD results in the formation of a new chromosome encoding *AM4* and *CGRP2* gene from proto-chromosome E (Figure 3). Additionally, duplication of proto-chromosome F gave rise to a new chromosome on which *AM3* gene is localized (Figure 3). The descendant of *AMY* gene in the vicinity of *AM3* gene might be lost (Figure 3). Although chromosome encoding *AM5* gene is likely to be duplicated, the counterpart of *AM5* gene has not been detected (Figure 3).

On the other hand, in the tetrapods lineage, *CGRP* gene of proto-chromosome E would tandemly duplicate and form new *CGRP*β gene (Figure 3). *CRSP* gene exists in the proximity of *CGRP*α gene on the same chromosome in the pig, horse, and dog (7, 73, 74). Dog *CRSP* gene resembles *CGRP*α gene in terms of generating CT mRNA by alternative splicing (73). These issues strongly support that *CRSP* gene is likely to be generated from *CGRP*α gene by tandem duplication and is an ortholog of *CGRP*β gene. Further, Tetrapods are likely to possess *AM2, AMY*, and *AM5* gene in the similar chromosomal position with proto-chromosome (Figure 3).

In cartilaginous fish, it is assumed that the chromosomal location of CT/CGRP family peptide gene is similar to that of CT/CGRP family peptide gene in ancestral proto-chromosome. Two *AM* genes have been identified in elasmobranch and holocephalans (9), and a partial CT sequence has been determined in stingray (8). AMY is identified from transcriptomic analysis using the lesser spotted shark (10). No report of *CGRP* gene from these fishes has been published. Thus, the search of CT/CGRP family peptide member is expected to be incomplete. Moreover, the chromosomal localization of identified CT/CGRP family peptide genes remains enigmatic. Comprehensive identification of CT/CGRP family peptide gene and their chromosomal localization using cartilaginous fish genome is necessary.

**Figure 4:**
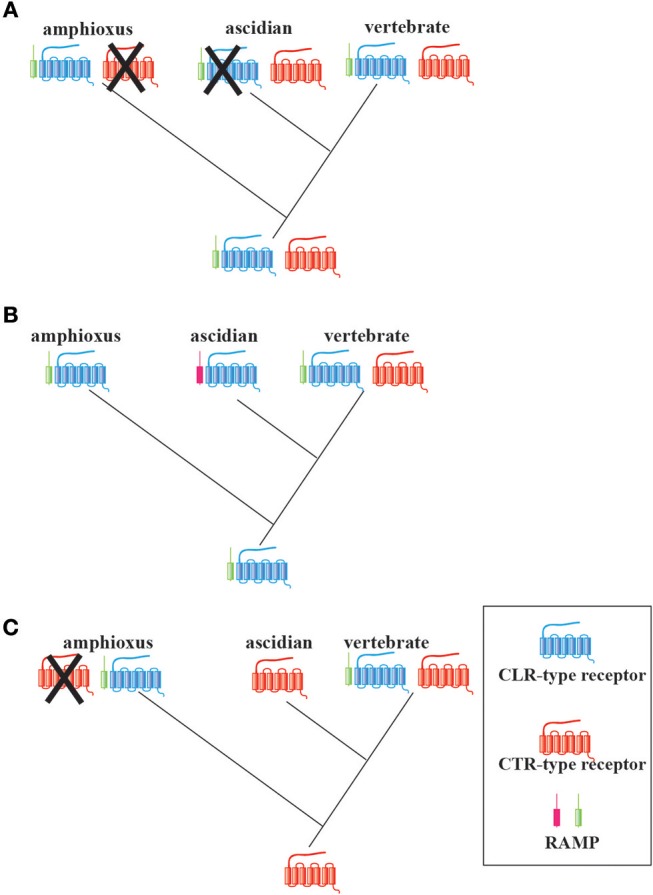
Three possible evolutionary scenarios of the CT/CGRP family receptors and RAMPs in chordates. **(A)** A common ancestor of chordates might have already possessed the RAMP-independent *CTR*-type gene, RAMP-dependent *CLR*-type gene, and *RAMP* gene. Gene loss of the *CTR*-type gene and *CLR*-type gene might have occurred in the amphioxus and ascidian lineage, respectively. **(B)** A primitive chordate might have had only a *CLR*-type gene and *RAMP* gene. Although an ascidian *RAMP*-like gene exists, this gene has not yet been identified. A *CTR*-type gene might have arisen in the vertebrate lineage. **(C)** A common ancestor of chordates might have possessed a *CTR*-type gene. *CTR*-type gene might have been lost in extant amphioxus. Furthermore, both amphioxus and vertebrates might have independently acquired the *RAMP*-like gene and *CLR*-type gene.

Next, I propose three possible evolutionary scenarios of CT/CGRP family peptide receptor from invertebrates to vertebrates (Figure 4). The first scenario presumes that the ancestral chordates already possessed both RAMP-independent *CTR*-type and RAMP-dependent *CLR*-type genes (Figure 4A). *CTR*-type gene can form an active receptor without heterodimer of RAMP, whereas *CLR*-type gene is not able to create an active receptor without RAMP. Subsequently, the *CTR*-type and *CLR*-type gene may have been lost in the amphioxus and ascidian lineage, respectively. This notion is in accordance with the previous findings that the amphioxus and ascidian peptide exhibit hybrid characteristics of CT and other family members and CT-like features, respectively. In turn, a common ancestor of vertebrates might have preserved *CTR-*type, *CLR-*type, and *RAMP* genes. The formation of CTR/CLR-RAMP complexes enabled the recognition of multiple CT/CGRP family peptides and the fulfillment of multiple functions of CT/CGRP family peptides in the vertebrate lineage. The second scenario speculates that the receptor type of a common ancestor of chordates only comprised a RAMP-dependent *CLR*-type gene (Figure 4B). Considering that the amino acid sequence similarity of Bf-RAMP-LPs with human RAMPs was low, the following possibility exists: although ascidians may possess a protein that acts as a RAMP-like escort protein for its CLR-type protein, this protein is not detected by homology search owing to low sequence similarity with vertebrate RAMPs. In turn, a RAMP-independent *CTR*-type gene might be newly acquired in the vertebrate lineage, which would assist vertebrates in responding to various CT/CGRP family peptides. The third scenario implies that a common ancestor of chordates carried a RAMP-independent *CTR*-type gene (Figure 4C). Amphioxus acquired the RAMP-dependent *CLR-*type gene and lost the *CTR*-type gene, whereas ascidians maintained the RAMP-independent *CTR*-type gene. In vertebrates, the *CLR*-type gene was newly generated from the *CTR*-type gene by gene duplication. To verify these hypotheses, evaluation of the ligand-receptor recognition and exploration of RAMP in Ambulacraria, which is a more primitive phylum than chordates, is required. Moreover, the molecular function of CTR/CLR in a primitive vertebrate such as cyclostome would further assist in evaluating the diversification process in the vertebrate CT/CGRP family system.

## Conclusions and perspectives

In vertebrates, the investigation of CT/CGRP family peptide and their receptor complexes has been intensively performed particularly in mammals. However, the knowledge of CT/CGRP family in cartilaginous fishes and cyclostomes is insufficient because comprehensive identification of CT/CGRP family peptide has not been completed yet. To describe a more precise evolutionary story of CT/CGRP family, further research of CT/CGRP family in cartilaginous fish and cyclostomes is necessary. On the other hand, data for invertebrate deuterostome CT/CGRP family peptides have been also accumulated. Particularly, in protochordates, molecular characterization and biological effects on vertebrates have been elucidated. These investigations demonstrate the similar characteristics between protochordate CT/CGRP family peptides and vertebrate CT/CGRP family peptides. Moreover, the ligand-receptor relationship and RAMP function have been clarified in an amphioxus. Notably, the molecular function of amphioxus CT/CGRP family receptors is not reminiscent of vertebrate CTR but rather of CLR. Moreover, RAMP functions are also conserved between amphioxi and vertebrates. However, no molecular functional data of receptors and RAMPs have been reported for *C. intestinalis*. Thus, further investigation of receptor function and evaluation of the existence of RAMP-like proteins are necessary. In the case of molecular function analysis of *Ciona* CT/CGRP family receptor, the *Ciona* CT/CGRP family receptor candidate has not been expressed in mammalian and insect cells. Therefore, it would be necessary to establish the primary culture and cell lines from *Ciona* individuals and utilize them in the forced expression system of *Ciona* CT/CGRP family receptor. Regarding the exploration of RAMP candidates, motif searches appear to represent an effective approach because chordate RAMPs show low primary amino acid sequence similarity and conserve structurally relevant motifs such as the single transmembrane domain and four Cys residues. Furthermore, the physiological activity of CT/CGRP family peptides in invertebrate chordates remains to be determined. Further clarification of receptor and physiological function in protochordates will likely provide insight into the evolutionary process of CT/CGRP family peptides from invertebrates to vertebrates.

## Author contributions

The author confirms being the sole contributor of this work and has approved it for publication.

### Conflict of interest statement

The author declares that the research was conducted in the absence of any commercial or financial relationships that could be construed as a potential conflict of interest.
